# Single-visit endodontic treatment under general anaesthesia in adult and adolescent patients with special needs: a systematic review

**DOI:** 10.1007/s10266-024-01030-z

**Published:** 2024-12-13

**Authors:** Shirin El-Sayed, Jelena Petrovic, Cornelia Frese, Caroline Sekundo

**Affiliations:** Department of Conservative Dentistry, Clinic for Oral, Dental and Maxillofacial Diseases, University Hospital Heidelberg, Heidelberg University, Im Neuenheimer Feld 400, 69120 Heidelberg, Germany

**Keywords:** Endodontic treatment, Root canal therapy, Special needs, General anaesthesia, Disability

## Abstract

To improve dental care for individuals with special needs, it is crucial to understand the effectiveness of endodontic treatments under general anaesthesia. This systematic review explores the feasibility, prognostic factors, and outcomes of root canal treatment and pulpotomy performed under general anaesthesia in adult and adolescent patients with special needs. A comprehensive search of Cochrane Library and MEDLINE databases was conducted until July 2024. The quality of evidence was assessed using the Newcastle–Ottawa scale (NOS). Among the 637 initially identified studies, 5 met the inclusion criteria. Treatment outcomes, radiographic findings, and clinical survival rates were evaluated. Root canal treatment success rates ranged from 81.5 to 90% over a minimum observation period of 12 months. Survival rates varied from 87.7% (9 year cumulative survival rate) to 89.8% (5 year survival rate). One study showed a lower failure risk in endodontic treatment under general anaesthesia compared to local anaesthesia. Another study found no significant difference in root canal treatment quality between general and local anaesthesia. Influencing factors identified by multivariate regression analyses included soft diet, periodontal status, oral hygiene status, pulp vitality, and length of root canal filling. Limited evidence suggests that endodontic treatment under general anaesthesia is feasible and can yield favourable outcomes in patients with special needs. However, the scarcity of studies and concerns about publication bias and methodological limitations emphasize the need for further research.

**Supplementary Information:**

The online version contains supplementary material available at 10.1007/s10266-024-01030-z.

## Introduction

Conservative dentistry offers various therapeutic options for the long-term preservation of teeth, including root canal treatment (RCT). RCT is a well-established procedure that has been investigated in multiple studies, providing substantial evidence of its effectiveness [1–4]. However, when it comes to managing patients with special needs, tooth extractions remain the preferred approach over RCT, due to potential challenges such as limited patient adherence and increased risk of complications [5, 6].

Given the estimated global population of 1.3 billion people with disabilities (16% of the global population) [7], patients with special needs are becoming more prevalent in dental care facilities. Individuals with disabilities have the right to receive an equal level of care as the rest of the community [8], addressing their unique requirements and circumstances. However, not all patients with special requirements can be treated regularly in a dental chair. In cases where treatment adherence is challenging, extensive dental procedures are commonly performed under general anaesthesia for this specific patient population. General anaesthesia requires specialized training and equipment. It is also associated with a certain degree of risk, such as airway obstruction, cardiovascular complications or allergic reactions. The risks may be higher in special needs patients due to their underlying medical conditions, their medication or anatomical variations. In addition to the need for trained experts such as anaesthesiologists, the utilization of general anaesthesia can result in logistical challenges particularly when inpatient facilities and resources from other departments are necessary. Its use can thus impose significant financial burdens on health care providers and families [9]. It is crucial that treatment under general anaesthesia remains as short as possible and provides a positive prognosis, minimizing the need for subsequent anaesthesia-based interventions. General anaesthesia for the purpose of RCT, which are often very time-consuming and may require several sessions, must, therefore, be critically questioned.

However, this topic has received limited attention from researchers to date. The aim of this paper is thus to review the existing literature and to assess the technical feasibility, prognostic factors and outcomes of RCT and pulpotomy performed under general anaesthesia in patients with disabilities and special needs.

The following questions were addressed:

1. Is single-visit RCT of adult and adolescent patients with special needs under general anaesthesia a feasible approach?
2. What are the outcomes of RCT in adult and adolescent patients with special needs under general anaesthesia in terms of success and survival?
3. Which factors influence the outcomes of RCT in adult and adolescent patients with special needs under general anaesthesia?
4. How does the quality of RCT under general anaesthesia compare to treatment under local anaesthesia in adult and adolescent patients with special needs?

To address the different objectives of the review, multiple PECO/PEO frameworks were employed. For comparing the quality and outcomes of root canal treatment under general anaesthesia versus local anaesthesia, the following PECO framework was used [10]:

Population (P): adult patients and adolescent with special needs

Exposure (E): root canal treatment or pulpotomy under general anaesthesia

Comparison (C): treatment under local anaesthesia

Outcome (O): periapical status or survival of the tooth without clinical signs of pathology

For the research question regarding prognostic factors, a PEO framework was chosen:

Population (P): adults and adolescents with special needs undergoing general anaesthesia

Exposure (E): different prognostic factors

Outcome (O): success and survival

## Materials and methods

### Protocol and registration

The systematic review adhered to the guidelines of the Preferred Reporting Items for Systematic Review and Meta-Analysis (PRISMA) [11] and was registered with PROSPERO under the registration number CRD42023430250.

### Search strategy

A comprehensive literature search was conducted by two examiners (CS, SES) using the Cochrane Library and MEDLINE databases (via Ovid), covering the literature from 1946 up to 11 July 2024. The references of the retrieved results were equally reviewed for further studies that might have been neglected during the initial search. The following search term was used in MEDLINE via Ovid and adapted for Cochrane Library:

(exp Periapical Diseases/ or exp Endodontics/ or exp dental pulp/ or exp dental pulp cavity/ or exp “tooth root”/ or exp “Root Canal Filling Materials”/or endodontic treatment.mp.or pulpotom*.mp.or pulpectom*.mp. or root canal treatment.mp.or dental trauma.mp.) and (Intellectual Disability/or disabled persons/or special need*.mp.or disabled.mp.or disabilit*.mp.or exp anesthesia, dental/ or exp anesthesia, general/ or handicap*.mp. or endotracheal intubation.mp. or Intubation, Intratracheal)and (exp Adult/ or adult*.mp.) and (exp Adolescent/or adolescent*.mp.)

### Eligibility criteria

The inclusion criteria specified that the articles had to be published in English or German. All study designs were considered eligible, except for case reports, letters, commentaries, editorials, reviews or abstracts. The study population was limited to adults and adolescents. The treatment must have been performed under general anaesthesia in patients with special needs, and the reported results must have included the outcome or quality assessment of the root canal treatment.

### Study selection and data extraction

Duplicate articles were eliminated and the reviewers independently screened the remaining articles based on their titles and abstracts. The second stage involved a full-text article screening process. In cases of disagreement, consensus was achieved among the reviewers through discussions and by seeking input from a third reviewer (CF).

The data extraction was performed by one author (SES) and later reviewed by a second author (CS). For those studies fulfilling the inclusion criteria, data extraction included information on study design, year of publication, mean age of the population, mean duration of general anaesthesia procedure, treatment approach, control group, if exists, outcome assessment, success rate, outcome definition and significant influencing factors (Tables 1, 4).

**Table 1:** characteristics of the included studies

Study	Study design	Year	Origin	Study population	Age (years)	Mean duration of GA	Treatment approach	Control (if exists)	Sample size (teeth)	Follow-up time	Outcome assessment and definition	Results
Alsaleh et al. [30]	Retrospective cross-sectional study	2012	France	Neuromotor disabilities, dental anxiety or phobia, dementia	GA: ♀28.5 ± 13.5 ♂28.6 ± 12.7	120 ± 38 min	RCT in GA single visit	RCT in LA multiple visits by dental students	GA: 255 LA: 247	NA	Radiographic findings, proportion of satisfactory RCQET	63% in both groups
Chen et al. [34]	Retrospective cohort study	2022	Taiwan	Intellectual disability, dementia, autism, chronic mental illness	GA: 25.3 ± 13.7 LA: 31.5 ± 16.5	NI	RCT in GA single visit	RCT in LA	GA: 280 LA: 217	9 Y	Clinical findings, 9 year cumulative survival rate	GA group: 87.7% (95% CI 77.3–99.4); Non-GA group: 74.5% (95% CI 68.1–81.5), Reduced risk of failure in GA group AHR for RCT failure in the GA group compared to the non-GA group: 0.24 (95% CI 0.12–0.49)
Chung et al. [33]	Retrospective cohort study	2019	Korea	Intellectual or cognitive disabilities, ASA 1–4	26.2 ± 14.1	NI	RCT in GA single visit	None	271	56.1 ± 27.9 M	Radiographic and clinical findings, periapical healing	81.50% after 56.1 ± 27.9 M
Cousson et al. [31]	Open cohort study	2014	France	Mental or cognitive deficiencies, dental fear or phobia, medically indicated, dementia	♀28.5 ± 10.8 ♂28.6 ± 10.3	113.4 ± 35 min	RCT or Pulpotomy single visit in GA	None	P: 32 RCT: 193	1-6 M (RCT *n* = 52, P *n* = 19) 6-24 M (RCT *n* = 64 P = 13) > 24 M (RCT *n* = 77, P *n* = 0)	Radiographic and clinical findings, success rates over different follow-up periods	Success: 1-6 M: RCT: 75%, *P*: 95%; 6-24 M: RCT: 88%, *P*: 100%; > 24 M: RCT: 90%; Overall: RCT: 85%, *P*: 97%; Total success: 87%
Chang et al. [32]	Retrospective cohort study	2017	Korea	Mental retardation, neurocognitive disorders, developmental disorders, dental phobia, Alzheimer’s disease, others	27 ± 14.1	NI	RCT in GA single visit	None	381	6–81 M	Clinical findings, 5 year survival rate	89.8 ± 3.4

*GA* general anaesthesia, *LA* local anaesthesia, *RCT* root canal treatment, *P* pulpotomy, *NI* no information, *M* months, *NA* not applicable, *RCQET* radiographic criteria for quality of endodontic treatment, ♀ females, ♂ males, *AHR* Adjusted hazard ratio

### Assessment of methodological quality

The Newcastle–Ottawa Scale (NOS) (for cohort and adapted for cross-sectional studies) [12, 13] was employed to assess the quality of the included studies. This scale evaluates the methodological quality of non-randomized and observational studies including cohort and case–control studies. It comprises three main domains: selection of study groups, comparability of groups, and ascertainment of exposure or outcomes. Each item in the scale is assigned a star if the study meets the criterion with a maximum possible score of 9 stars for the highest quality. Higher scores indicate better methodological quality and lower risk of bias. Studies were classified as low quality (0–4 stars), moderate quality (5–6 stars), and high quality (more than 7 stars) [14]. The quality assessment was conducted independently by two reviewers, with a *k* value of 1.0 [15].

A comprehensive meta-analysis was not feasible due to the limited number of eligible studies and heterogeneity in outcome measures and follow-up-periods.

## Results

### Study selection

**Fig. 1:**
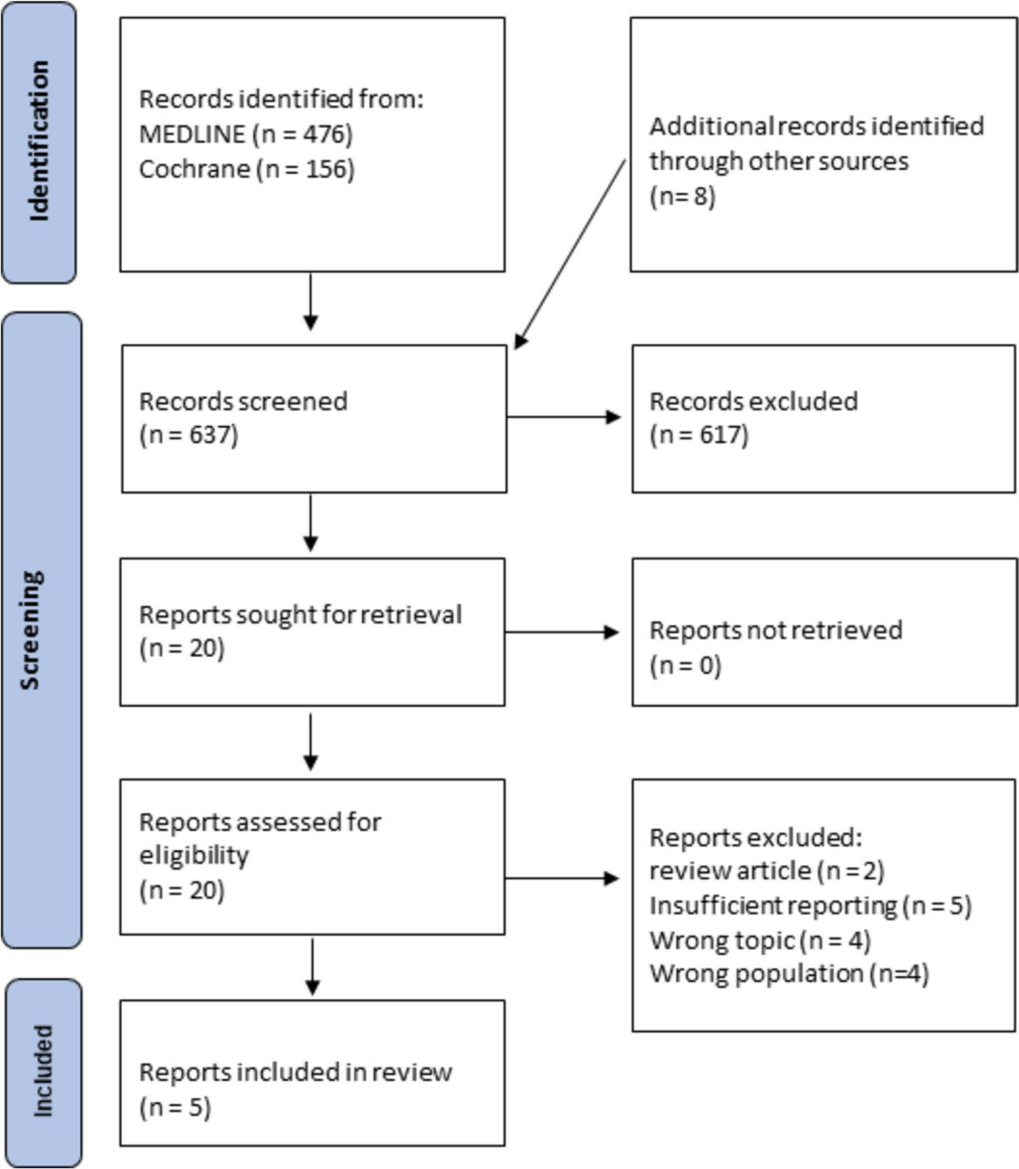
PRISMA flowchart with study selection process

The initial search retrieved 637 studies. 20 studies underwent full text screening of which only 5 studies met the inclusion criteria according to the PECO/PEO framework. Among the 15 excluded studies, three studies were epidemiological studies [16–18] and two were review articles [19, 20]. The remaining 10 studies were excluded for various reasons: incorrect population (children) [21–24], absence or insufficient data about the outcome of endodontic treatment [25–28], endodontic treatment performed in local anaesthesia with insufficient data about the general anaesthesia cohort [29] and questionnaire-based studies [30]. The flowchart depicted in Fig. 1 illustrates the study selection process based on the PRISMA guidelines.

### Study characteristics

Table 1 provides detailed information about the characteristics of the included studies. No randomized controlled trials could be identified. All studies collected retrospective data and were published between 2012 and 2022. The countries of origin were France [31, 32], Korea [33, 34] and Taiwan [35]. The reasons for treatment under general anaesthesia in the included studies were compromised neurological conditions, cognitive disabilities, dental anxiety or phobia, medical indications or unknown. All of the studies were observational in their design, with three being retrospective cohort studies [33–35], one being an open cohort study [32] and one cross-sectional study with retrospective data collection [31].

Four studies assessed the outcome of root canal treatment of special needs patients under general anaesthesia [32–35] with only two of them considering radiographic follow-up to determine success and survival rates [32, 34]. One study evaluated the radiographic quality of root canal fillings under general anaesthesia and local anaesthesia [31]. The mean age was comparable in all studies and ranged from 25.3 to 28.6 years. All tooth types, including anterior teeth, premolars, and molars, were represented in all studies. The mean follow-up time varied among the studies. Two studies included a control group that received treatment under local anaesthesia [31, 35]. Two of the included studies provided information about the duration of the operation. Cousson et al. [32] registered a mean duration of 113.4 ± 35 min, with a mean of 12.8 ± 5.3 items of procedures per intervention. Alsaleh et al. [31] had similar durations of 120 ± 38 min, with a mean of 11.6 ± 5.5 items. All studies provided detailed descriptions of the endodontic treatment procedures performed except for Chen et al. [35]. Table 2 provides an overview of the technical information of the RCTs. The definition of success and survival varied. Cousson et al. [32] classified outcome as “success,” “uncertain,” or “failure” based on the periapical index (PAI), with success defined by a stable or improved PAI, “uncertain” by minor PAI increases or stability and failure by an increased PAI In the study by Chung et al. [34], “healed” indicated complete resolution of radiolucencies and no symptoms; “uncertain” showed incomplete resolution and “not healed” showed no reduction in periapical radiolucencies. Chen’s study [35] assessed the cumulative survival rate of teeth using Kaplan–Meier analysis, focusing on the absence of RCT failure (need for re-treatment or extraction) without radiographic evaluation. Chang et al. [33] defined success as the absence of the need for further intervention to maintain tooth function and survival as the need for intervention with extraction considered a failure. Alsaleh et al. [31] evaluated the radiographic quality of root canal fillings under general and local anaesthesia. A satisfactory RCT outcome required the root filling to extend within 2 mm of the apex, be free of voids, and have all visible canals thoroughly filled.

**Table 2:** descriptions of the endodontic treatment procedures

Study	Rubber dam	Working length determination	Irrigation solutions	Cleaning and shaping	Obturation technique	Restoration materials
Alsaleh et al. [30]	~ 80% of cases	Preoperative argentic radiograph or apex locator or no determination	2.5% NaOCl and EDTA	Crown-down technique with hand files and sequential rotary instrumentation	Master cone of gutta-percha with ZOE sealer lateral compaction thermomechanical compaction	Composite, amalgam, GIC, crowns
Chen et al. [34]	No information	No information	No information	No information	No information	No information
Cousson et al. [31]	~ 92% of cases	Argentic radiograph apex locator	2.5% NaOCl	Crown-down technique with hand files and sequential rotary instrumentation	Thermomechanical compaction with ZOE sealer	Composite, GIC bonded amalgams, stainless steel crowns
Chung et al. [33]; Chang et al. [32]	Yes	Apex locator digital intraoral radiography	2.6% NaOCl	Engine-driven instruments stainless steel K-files	Gutta-percha cones with epoxy-resin sealer continuous wave condensation	Adhesive resin, flowable composite, composite resin

*ZOE* zinc oxide eugenol, *GIC* glass ionomer cement

### Methodological quality assessment

The NOS quality assessment for the five studies provided varying levels of methodological quality. The overall ratings ranged from six to eight stars with differences in selection, comparability, and outcome assessment criteria. Alsaleh et al. [31] received six stars, showing strengths in the selection of cohorts and outcome assessment but lacked comparability due the absence of clear evidence of controlling for key confounders and did not include a sample size calculation. Chen et al. [35], Chung et al. [34] and Chang et al. scored the highest with eight stars, showing strong selection criteria and good control for confounders, along with adequate follow-up. Chang et al. [33] and Chung et al. [34] lacked a non-exposed cohort and had moderate comparability. Cousson et al. [32] received seven stars. The study demonstrated strong selection and outcome assessment but had moderate comparability. Appendix S1 provides detailed explanations and justifications for the quality assessment of each study. The methodological quality of the studies is presented in Table 3.

**Table 3:** Newcastle–Ottawa Scale assessment of methodological quality

Study	Study design	Selection	comparability	Outcome	Overall
Alsaleh et al. [30]	Cross-sectional study with retrospective data collection	☆☆☆☆	–	☆☆	☆☆☆☆☆☆ moderate
Chen et al. [34]	Retrospective cohort study	☆☆☆☆	☆☆	☆☆	☆☆☆☆☆☆☆☆ high
Chung et al. [33]	Retrospective cohort study	☆☆☆	☆☆	☆☆☆	☆☆☆☆☆☆☆☆ high
Cousson et al. [31]	Open cohort study	☆☆☆	☆	☆☆☆	☆☆☆☆☆☆☆ high
Chang et al. [32]	Retrospective cohort study	☆☆☆	☆☆	☆☆☆	☆☆☆☆☆☆☆☆ high

### Qualitative synthesis

Cousson et al. [32].documented a success rate of 90% for the RCT subgroup over 24 months. They examined 32 pulpotomies, with only 13 teeth being followed up for a period of 6–24 months with a success rate of 100%. The overall success rate for RCT was 85% with 10% of cases deemed uncertain and 5% classified as failures. Seven teeth with RCTs were extracted with six of these extractions unrelated to endodontic complications. Among the 271 cases included in the outcome analysis by Chung et al. [34], 221 (81.5%) achieved complete healing while 43 cases (15.9%) were classified as uncertain due to incomplete resolution of radiolucent signs. Only 7 cases (2.6%) were considered failures. Chen et al. [35] reported a 9 year cumulative survival rate of 87.7% (95% CI 77.3–99.4) for GA and 74.5% (95% CI 68.1–81.5) for LA., while Chang et al. reported 5 year survival rate was 89.8 ± 3.4 and the 5 year success rate was 86.4 ± 4. The results of Alsaleh et al. [31] indicated no differences in the number of teeth meeting all quality criteria persisting of obturation length, obturation density and complete filling of the canal system. However, when considering each criterion separately, teeth treated under GA demonstrated significantly better results regarding root filling density than those treated in LA (93% vs. 80%).

Potential influencing factors were examined across the included studies with varying findings. Alsaleh et al. [31] investigated the impact of tooth type (specifically molars) and periapical status (PAI > 2) on RCQET performed under GA. It found a significant effect on the outcome, with molars and teeth with higher PAI demonstrating a negative impact on the radiographic quality of the root canal filling. The analysis lacked adjustment for confounding effects. In Chen’s study [35] factors such as the use of GA, tooth type, and pre-existing conditions were examined. The risk of failure for RCT was assessed and both crude and covariate-adjusted analyses were conducted. Their findings revealed a significantly reduced risk for the GA group with an adjusted hazard ratio of 0.24 (95% CI 0.12–0.49), indicating that GA positively influenced the survival rates of RCTs. The study found no significant association between comorbidities and increased risk of RCT failure. Chung et al. [34] investigated several factors, including diet type (soft, GI intubation, liquid), poor oral hygiene maintenance, non-vital pulp, and the length of root filling (either short or over-filled). Patients consuming a softer diet were 67% less likely to show complete periapical healing (OR = 0.33) and those with poor oral hygiene had a 70% lower likelihood of healing (OR = 0.30). Nonvital pulpal conditions and inadequate root filling length were negatively related to periapical healing with statistically significant impacts. The study used multivariate regression analysis to adjust for these factors. Cousson et al. [32] explored tooth type, pulpal status, level of endodontic difficulty, periapical status and the technical quality of RCT. The study found that none of these examined factors influenced the success of the endodontic treatments. Chang et al. [33] examined age (> 40), non-parental caregiver presence, low cooperation, periodontal disease and soft diet. They found that these factors negatively influenced the outcome of endodontic treatments. The study also observed a trend suggesting a positive impact of having a low DMFT (Decayed, Missing, and Filled Teeth) score, the presence of non-vital teeth, and crown restorations, although this trend did not reach statistical significance. The analysis included adjustments for these factors. Table 4 provides an overview of the significant factors.

**Table 4:** contribution and significance of influencing factors

Study	Influencing factors	Adjustment
Alsaleh et al. [30]	Tooth type (molars) (−)* Periapical status PAI > 2 (−)*	X
Chen et al. [34]	General anaesthesia (+) *	✓
Chung et al. [33]	Diet type (soft, GI, liquid) (−)* Poor oral hygiene maintenance (−)* Non-vital pulp (−) * Short or over-filled root filling length (−)*	✓
Cousson et al. [31]	None of the examined factors had an impact on the outcome	X
Chang et al. [32]	Age (> 40) (−)* Non-parental caregiver (−)* Low cooperation (−) * PERIODONTAL disease (−)* SOFT diet (−)* High DMFT (−) Vital tooth (−) Crown restoration (+)	✓

*significant influence; ( +) positive influence on the outcome; (−) negative influence on the outcome

Adjustment: statistical adjustments for potential confounding factors were applied (✓) or not applied (X)

## Discussion

This systematic review aimed to assess the technical feasibility, prognostic factors, and outcomes of RCT and pulpotomies performed under general anaesthesia in adult patients with disabilities and special needs. Five studies could be identified that met the inclusion criteria according to the PECO/PEO framework. Two of the four key questions examined were related to the feasibility and outcomes of single-visit RCT. The available evidence suggests that RCT under GA is a feasible approach for adult and adolescent patients with special needs, resulting in an enhanced oral health related quality of life [30].

RCT was completed in a single-visit setting in all studies. The literature suggests that there is no difference in the success rates between single-visit and multiple-visit therapy [36]. Paredes-Vieyra et al. reported success rates of 96% and 89% for single-visit and two-visit therapy of teeth with apical periodontitis, respectively [37]. A recent review investigating the effectiveness of intracanal dressing in a multiple visit-approach concluded that single-visit treatment is associated with better outcomes [38].

The reported success and survival rates of RCT under GA as well as the follow-up periods varied among the included studies. It should be noted, however, that the clinical follow-up of these special needs patients is much more complex and may differ from the regular procedure in clinical studies. Cousson et al. [32] reported a success rate of 90% for root canal treatments over 24 months, while Chung et al. [34] reported a success rate of 81.5% for a mean follow-up time of 56.1 ± 27.9 months. Chen et al. [35] found a 9 year cumulative survival rate of 87.7% for GA-treated patients, but radiographic follow-up was not available to evaluate the success rate. These variations in reported outcomes may be attributed to differences in follow-up periods and definitions of success and survival. Additionally, the small sample sizes of the included studies may have influenced the reported success rates. The reported findings are nevertheless comparable to those reported in the literature for treatment under LA. Burns et al. documented a weighted pooled success rate of 82% (95% CI 79.3–84.8%) under strict criteria in a recent review [1]. Ng et al. reported a success rate of 83% in a prospective study [4] and a weighted pooled survival rate of 87% (95% CI 82%, 92%) over a period of 8–10 years [3]. The results of the Toronto study phase 4 showed a success rate of 86% 4–6 years after treatment for the pooled sample of phase 1–4 [2].

The data on pulpotomies provided by Cousson et al. [32] were limited. None of the teeth were included in the follow-up group beyond 24 months. The success rate was 100% for 13 teeth, which were examined after 6–12 months.

Regarding the third query, several factors were identified as potential influencers of the outcomes of RCT under GA in patients with special needs. Age, non-parental caregiver, low cooperation, periodontal disease, diet type, oral hygiene maintenance, pulp vitality, and root filling length were among the factors investigated. Pulp vitality and root filling length are well-documented influencing factors in the available literature. Root canals obturated within 0–2 mm from the apex exhibited the most favourable outcomes [39–41].

Various studies have also shown that pulp vitality is a significant influencing factor [42]. In terms of periodontal disease as a significant influence factor, Ruiz et al. reported a 5.19-fold higher risk for apical periodontitis in the presence of periodontal attachment loss [43]. Khalinghinejad et al. reported a 1.9-fold likelihood for tooth loss after RCT for teeth with mild periodontal destruction and a 3.1-fold likelihood for teeth with moderate disease [44]. A soft diet seems to have a negative impact on the outcome, which may be related to the severity of the disability, making oral maintenance and hygiene difficult. Endodontically treated teeth also tend to have higher plaque accumulation [45].

The fourth question aimed to investigate potential differences in the quality of RCT under GA compared to treatment under LA, which was assessed in one of the included studies. Alsaleh et al. [31] found no significant differences in the overall quality of root canal treatments performed under GA and LA. This suggests that, despite the challenges posed by GA, the quality of RCT can still be achieved at a comparable level to treatment under LA. However, when evaluating specific criteria, teeth treated under GA demonstrated significantly better results in terms of root filling density. Under LA, patients may exhibit movement, reflexes, or sensitivity, which can make it more difficult for the practitioner to achieve optimal root canal filling density. In contrast, when patients are under GA, they are unconscious and do not exhibit such behaviours, allowing the practitioner to perform the root canal treatment with better control and precision. The improved ability to achieve higher root canal filling density under GA may result in better sealing of the root canal system, reducing the likelihood of bacterial recontamination and improving the long-term success of the treatment. The study presents several evident confounding factors that impact the validity of its conclusions. The treatment under LA in this specific study was performed by dental students, which might have affected the density of root canal fillings. The number of visits required for treatment differed between groups, with the GA group receiving treatment in a single visit and the LA group requiring multiple visits. This difference in treatment protocol could affect the success rates and patient compliance, potentially affecting the results in favour of one group over the other. The examined confounding factors tooth type and periapical status showed significant influence. However, the authors did not make any statistical adjustments to account for these variables. Overall these confounding factors suggest that the results of the Alsaleh study might not be entirely reliable and should be interpreted with caution. Chung et al. [34] found no correlation between the difficulty of the treated tooth based on the difficulty assessment of the American Association of Endodontics and the outcome [46]. However, case difficulty seems to influence the operating time [26]. The difficulty assessment form can serve as an additional tool in the decision-making process for treatment planning under GA.

Four of the five reviewed studies described the treatment procedure. Cousson et al. [32] and Alsaleh et al. [31] reported cases where compromises had to be made. For instance, the placement of rubber dam or determination of working length had to be neglected in some cases.

All studies employed sodium hypochlorite as an irrigant. Sodium hypochlorite is the most recommended irrigant in endodontics and has demonstrated high efficacy against anaerobic and facultative microorganisms. It possesses the ability to neutralize lipopolysaccharides and dissolve both vital and necrotic tissue [47, 48].

Overall, this systematic review provides a preliminary understanding of endodontic treatment under GA in adult patients with special needs. The included studies reported successful outcomes of root canal treatment and pulpotomy procedures performed under GA. It indicates that performing endodontic treatment under GA could be a viable option for this population. However, it is crucial to acknowledge certain limitations when interpreting the results.

A scientific limitation of this systematic review is the restriction of the literature search to only two languages, German and English. This language limitation may have resulted in the exclusion of relevant studies published in other languages, potentially leading to a bias in the findings. This constraint is due to the lack of language expertise required to evaluate studies in other languages. However, English is the predominant language of scientific research, ensuring consistency in the review process and allowing for a more feasible and standardized scope.

The potential presence of publication bias raises concerns regarding the reliability of the reported outcomes. The NOS quality assessment revealed certain limitations across the included studies. A moderate risk of bias was identified in the domains of patient selection and confounding variables. These findings suggest potential challenges in appropriately accounting for confounders and potential biases in how participants were selected. The results of these studies should therefore be interpreted with caution, taking into consideration these methodological limitations and the impact they may have on the validity of the findings.

The available evidence is also restricted to retrospective studies that do not include randomized controlled trials. Lack of eligible studies and heterogeneity of outcome measures prevented a comprehensive meta-analysis. Differences in sample size, follow-up duration, outcome definitions and confidence intervals across studies posed a challenge for pooling data meaningfully, despite the moderate to high quality of the included studies.

It is crucial to identify the optimal approach for endodontic treatment of patients with special needs under GA. This would improve clinical outcomes and reduce treatment duration and costs. The development of guidelines for decision making for or against tooth preservation by endodontic treatment to aid practitioners in decision-making would be beneficial.

## Conclusions

The presented review emphasizes the need for further research to determine the feasibility and outcomes of RCT under GA. Future studies should include randomized controlled trials with larger sample sizes and, if feasible in this collective, longer follow-up periods.

## Supplementary Information

Below is the link to the electronic supplementary material.

Supplementary file1 (DOCX 17 KB)

## Data Availability

Data available upon reasonable request from the authors.
