# Supplementary material for: Single-visit endodontic treatment under general anaesthesia in adult and adolescent patients with special needs: a systematic review
Source: Odontology. 2024 Dec 13;113(2):531–41. doi: 10.1007/s10266-024-01030-z (PMC11950014; doi:10.1007/s10266-024-01030-z)
Supplement: Supplementary file 1 — Supplementary file1 (DOCX 17 KB) [file 10266_2024_1030_MOESM1_ESM.docx]

| Study | Category | Item | Rating | Justification |
| --- | --- | --- | --- | --- |
| Alsaleh et al. | Selection *** | Representativeness of the Exposed Cohort | * | Somewhat representative of the average special needs patients requiring dental treatments under general anaesthesia in the community, no demographic information |
|  |  | Sample Size |  | did not include sample size calculation |
|  |  | Non-respondents |  | N.A. |
|  |  | Ascertainment of the exposure | ** | hospital records only |
|  | Comparability |  | - | no clear evidence of controlling for key confounders |
|  | Outcome *** | Assessment of outcome | ** | Radiographs and medical reports |
|  |  | Statistical Test | * | Appropriate statistical tests |
|  | Overall quality assessment |  | ****** | moderate |

| Study | Category | Item | Rating | Justification |
| --- | --- | --- | --- | --- |
| Chen et al. | Selection **** | Representativeness of the Exposed Cohort | * | Somewhat representative of the average special needs patients requiring dental treatments under general anaesthesia in the community, no demographic information |
|  |  | Selection of the non exposed cohort | * | Both the exposed (GA) and non-exposed (non-GA) cohorts were derived from the same hospital |
|  |  | Ascertainment of exposure | * | The exposure was ascertained using secure medical records |
|  |  | Demonstration that outcome of interest was not present at start of study | * | only first-visit RCT records were included |
|  | Comparability ** | basis of the design or analysis | ** | performed statistical adjustments for several key confounders in their analysis, used Cox proportional hazards models to estimate the hazard ratios for RCT failure |
|  | Outcome ** | Assessment of outcome | * | used record linkage to identify the outcomes of interest |
|  |  | follow-up long enough | * | minimum follow-up period of one year and extended up to nine years |
|  |  | Adequacy of follow up of cohorts |  | No statement |
|  | Overall quality assessment |  | ******** | high |

| Study | Category | Item | Rating | Justification |
| --- | --- | --- | --- | --- |
| Chung et al. | Selection *** | Representativeness of the Exposed Cohort | * | Somewhat representative of the average special needs patients requiring dental treatments under general anaesthesia in the community, no demographic information |
|  |  | Selection of the non exposed cohort |  | no explicit mention of a non-exposed cohort |
|  |  | Ascertainment of exposure | * | The exposure was ascertained using secure medical and radiographic records |
|  |  | Demonstration that outcome of interest was not present at start of study | * | teeth were treated for the first time during the study period |
|  | Comparability ** | basis of the design or analysis | ** | The study controls for multiple important factors using multivariate regression analysis |
|  | Outcome *** | Assessment of outcome | * | x-rays, medical records |
|  |  | follow-up long enough | * | follow-up period longer than 12 months |
|  |  | Adequacy of follow up of cohorts | * | description provided of those lost |
|  | Overall quality assessment |  | ******** | high |

| Study | Category | Item | Rating | Justification |
| --- | --- | --- | --- | --- |
| Cousson et al. | Selection *** | Representativeness of the Exposed Cohort | * | Somewhat representative of the average special needs patients requiring dental treatments under general anesthesia in the community, no demographic information |
|  |  | Selection of the non exposed cohort |  | no explicit mention of a non-exposed cohort |
|  |  | Ascertainment of exposure | * | The exposure was ascertained using secure medical records and radiographs |
|  |  | Demonstration that outcome of interest was not present at start of study | * | begins follow-up at the time of the GA session |
|  | Comparability * | basis of the design or analysis | * | The study controls for factors such as follow-up duration, pulpal status, periapical status, and technical quality of root canal treatment; no adjustment made |
|  | Outcome *** | Assessment of outcome | * | clinical and radiographic examinations |
|  |  | follow-up long enough | * | The follow-up period varied with some cases followed for up to two years |
|  |  | Adequacy of follow up of cohorts | * | detailed information on follow-up |
|  | Overall quality assessment |  | ******* | high |

| Study | Category | Item | Rating | Justification |
| --- | --- | --- | --- | --- |
| Chang et al. | Selection *** | Representativeness of the Exposed Cohort | * | Somewhat representative of the average special needs patients requiring dental treatments under general anaesthesia in the community, no demographic information |
|  |  | Selection of the Non-Exposed Cohort |  | the study does not mention a non-exposed cohort |
|  |  | Ascertainment of Exposure | * | The exposure was ascertained using secure medical records and radiographs |
|  |  | Demonstration that Outcome of Interest was Not Present at Start of Study | * | the outcomes of were only assessed after the treatment was performed |
|  | Comparability ** | Comparability of Cohorts on the Basis of the Design or Analysis | ** | The study performed statistical adjustments for several key confounders in their analysis, such as age, caregiver type, cooperation level, and periodontal disease using multivariate analysis models |
|  | Outcome*** | Assessment of Outcome | * | Outcomes were assessed using clinical and radiographic records |
|  |  | Was Follow-Up Long Enough for Outcomes to Occur | * | The follow-up period ranged from 6 to 81 months |
|  |  | Adequacy of Follow Up of Cohorts | * | detailed follow-up information with a significant portion of the cohort being followed up adequately |
|  | Overall quality assessment | Overall | ******** | high |
